# Where Does Metformin Stand in Modern Day Management of Type 2 Diabetes?

**DOI:** 10.3390/ph13120427

**Published:** 2020-11-27

**Authors:** Ehtasham Ahmad, Jack A. Sargeant, Francesco Zaccardi, Kamlesh Khunti, David R. Webb, Melanie J. Davies

**Affiliations:** 1 Diabetes Research Centre, University of Leicester, Leicester LE5 4PW, UK; jack.sargeant@leicester.ac.uk (J.A.S.); frazac@fastwebnet.it (F.Z.); kk22@leicester.ac.uk (K.K.); david.webb@uhl-tr.nhs.uk (D.R.W.); melanie.davies@uhl-tr.nhs.uk (M.J.D.); 2 NIHR Leicester Biomedical Research Centre, University Hospitals of Leicester NHS Trust and the University of Leicester, Leicester LE5 4PW, UK; 3 NIHR Applied Research Collaborations (ARC), East Midlands, Leicester LE5 4PW, UK

**Keywords:** metformin, type 2 diabetes, cardiovascular outcomes, atherosclerotic cardiovascular disease, glucose-lowering therapy, cardioprotection

## Abstract

Metformin is the most commonly used glucose-lowering therapy (GLT) worldwide and remains the first-line therapy for newly diagnosed individuals with type 2 diabetes (T2D) in management algorithms and guidelines after the UK Prospective Diabetes Study (UKPDS) showed cardiovascular mortality benefits in the overweight population using metformin. However, the improved Major Adverse Cardiovascular Events (MACE) realised in some of the recent large cardiovascular outcomes trials (CVOTs) using sodium-glucose co-transporter 2 inhibitors (SGLT2i) and glucagon-like peptide-1 receptor agonists (GLP-1RA) have challenged metformin’s position as a first-line agent in the management of T2D. Many experts now advocate revising the existing treatment algorithms to target atherosclerotic cardiovascular disease (ASCVD) and improving glycaemic control as a secondary aim. In this review article, we will revisit the major cardiovascular outcome data for metformin and include a critique of the UKPDS data. We then review additional factors that might be pertinent to metformin’s status as a first-line agent and finally answer key questions when considering metformin’s role in the modern-day management of T2D.

## 1. Introduction

Almost 60 years after it was first introduced for the treatment of type 2 diabetes (T2D), metformin (1,1-dimethylbiguanide hydrochloride) remains the most widely prescribed oral glucose-lowering therapy (GLT) for the management of T2D worldwide [1]. Metformin is endorsed by most clinical guidelines after the UK Prospective Diabetes Study (UKPDS) first demonstrated long-term metabolic benefits and reduced cardiovascular risk with metformin therapy in addition to less weight gain and fewer hypoglycaemic episodes compared to therapy with insulin and sulfonylurea (SU) [2]. Subsequently, the International Diabetes Federation (IDF) approved metformin as a first-line agent for management of T2D in 2005 in their first global guideline [3].

In 2011, the World Health Organisation (WHO) included metformin in its list of essential medicines along with SU and insulin [4], endorsing the important role metformin plays in the management of individuals with T2D globally. Metformin prescribing peaked from 55.4% (95% Confidence Interval (CI) 55.0, 55.8) in 2000 to 83.6% (95% CI 83.4, 83.8) in 2013 among all individuals with T2D who were on at least one medication for their diabetes management in the UK [5]. Similarly, in the USA first line use for metformin increased from 60% in 2005 to 77% in 2016 [6], reflecting the impact of national and international prescribing guidelines.

The pre-eminent role of metformin is based on decades of experience showing its safety and efficacy in most clinical settings with no associated weight gain (weight loss in some cases) or increased risk of hypoglycaemia, convenient dosing schedules, low cost, and global availability.

Over the past few years, we now have evidence of several other beneficial roles of metformin including diabetes prevention, use in gestational diabetes and polycystic ovarian syndrome (PCOS). The speculative actions of metformin with experimental evidence include reduced fasting hyperinsulinaemia and inflammation [7], modest improvement in lipid profile [8], anti-thrombotic [9] and anti-atherosclerotic potential [10], beneficial impact on gut microbiome and immune function [11] and a role in endothelial cell function [12,13]. All these effects could underpin improved cardiovascular outcomes in individuals taking metformin. A possible role in protection against certain types of cancers [14], cognitive disorders [15] and potential anti-ageing effects [16] has opened another debate beyond management of hyperglycaemia. This is explained by metformin’s diverse modes of action, some of which have not been fully explored.

In this review article, we examine the major cardiovascular outcome data for metformin including the UKPDS. We will also look at some of the additional benefits of metformin use over its competitors, which make it a convenient first choice. Finally, we will answer some key questions that require attention before we consider “dethroning” metformin.

**Figure 1:**
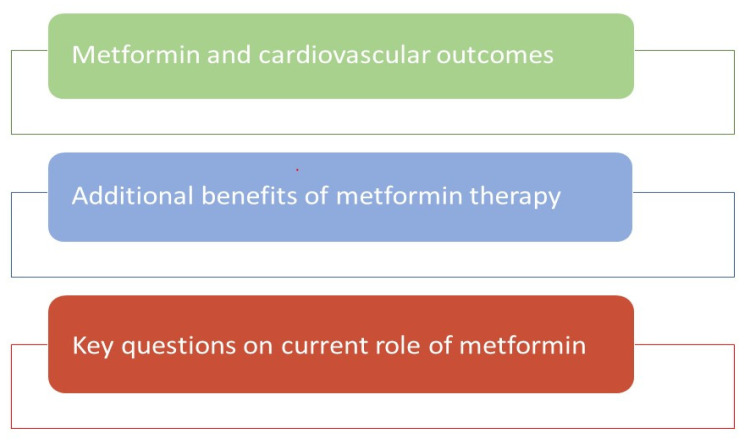
Summary diagram of individual sections.

We have divided this review article into three different sections for convenience of the readers as shown in Figure 1 below.

## 2. Metformin and Cardiovascular Outcomes

### 2.1. Metformin and the UKPDS, What Did It Tell Us?

Diabetes is an independent risk factor for the development of cardiovascular disease (CVD) [17]. CVD is the most prevalent macrovascular complication associated with diabetes and is the leading cause of death in this population [18,19]. Consequently, there has been much interest in exploring the effects of licensed glucose-lowering agents on CVD.

The first landmark study to show that metformin use was associated with reduced atherosclerotic cardiovascular disease (ASCVD) was the UKPDS [2]. A sub-study of this large multicentre trial in newly diagnosed individuals with T2D showed that compared to SU and insulin, metformin was associated with a reduction in any diabetes-related endpoint including myocardial infarction (MI) and heart failure (HF) (*p* = 0.0034), all-cause mortality (*p* = 0.021) and stroke (*p* = 0.032) over a mean duration of 10.7 years. Similarly, compared to the conventional group (diet-control only), metformin therapy resulted in a risk reduction (RR) of 32% (95% CI 13, 47; *p* =0.002) for any diabetes-related endpoint (microvascular or macrovascular), 42% (95% CI 9, 63; *p*= 0.017) for a diabetes-related death composite comprising of MI, peripheral vascular disease (PVD) and stroke and 35% (95% CI 9, 55; *p* = 0.011) for all-cause mortality. Although, there was also a trend towards reduced microvascular endpoints with metformin therapy, however it did not reach statistical significance. The UKPDS study group concluded that metformin might be considered the first-line pharmacological therapy of choice in overweight individuals with T2D as it appeared to reduce risk of diabetes-related endpoints in these individuals along with less weight gain and fewer hypoglycaemic episodes compared to insulin and SU [2]. The observed cardiovascular benefits of metformin were not explained entirely based on glycaemic control.

A 10-year post-trial monitoring of the UKPDS cohort showed that in the metformin group, significant RR persisted for any diabetes-related end-point (RR 0.79, 95% CI 0.66, 09.5; *p* = 0.01), MI (RR 0.67, 95%CI 0.51, 0.89; *p* = 0.005), and death from any cause (RR 0.73, 95% CI 0.59, 0.89; *p* = 0.002) even though between-group differences in glycated haemoglobin (HbA1c) were lost 1-year after completion of the main trial [20]. This lead to the concept of “legacy effect” or “glycaemic memory” suggesting that putative long-term benefits of intensive early glycaemic control in newly diagnosed individuals with T2D persist even if followed by a return to “usual” less intense care [21].

### 2.2. What are the Main Criticisms on the UKPDS Data?

#### 2.2.1. Possible Flaws in the Design of UKPDS

The results of the UKPDS data highlighting the benefits of metformin have been challenged for various reasons including;

- lack of blinding as the conventional group was not administered a placebo,
- change in significance threshold from initially chosen *p* < 0.01 to *p* < 0.05 during the study increasing the probability of results being due to chance alone and
- long period of follow-up leading to risk of attrition bias and difficulty in maintaining the comparability between the groups [22].

After UKPDS, an observational Danish study published in 2011 compared metformin to insulin secretagogues (SU and repaglinide) looking at all-cause mortality, cardiovascular mortality, and the composite of MI, stroke, and cardiovascular mortality in patients with or without previous MI [23]. Monotherapy with individual insulin secretagogues was associated with increased mortality and cardiovascular risk compared to metformin. A previous study published in 2005 to estimate the congestive heart failure (CHF) risk associated with specific therapies for diabetes showed lowest incidence of CHF in those taking metformin compared to other oral and injectable therapies [24]. Further consolidating the UKPDS findings were the results of the Diabetes Audit and Research in Tayside Scotland (DARTS) study published in 2006, which looked at prescribing database for population of Tayside, Scotland [25]. Of the 5730 study patients newly treated with one or more oral GLTs, those treated with metformin had lower risk of adverse cardiovascular outcomes than those treated with SU only, or combinations of SU and metformin. Although not all data has been very convincing, these observational studies support the notion that the findings of UKPDS were not merely by chance alone.

#### 2.2.2. Participant Characteristics

One main criticism is that UKPDS only comprised of individuals with a new diagnosis of T2D not based on a tight criteria (fasting blood glucose range from 6.1 to 15.0 mmol/L) who were not at particularly high risk of CVD in the first place [26]. It is important to remember that the inclusion of obese individuals in UKPDS is consistent with general T2D population and is more representative of the current diabetes population. Later studies indicate that metformin has beneficial effects on CVD even in those with established risk factors or in those with longer duration of diabetes. In data looking at individuals with T2D and established acute coronary syndrome (ACS), metformin users were found to have a lower all-cause mortality rate (hazard ratio (HR) 0.50, 95% CI 0.26, 0.95; *p* = 0.0346) in the primary analysis compared to non-metformin users [27]. A large trial of 390 individuals treated with insulin investigated whether metformin addition has sustained beneficial metabolic and cardiovascular effects compared to placebo in individuals with T2D [28]. The primary outcome was a composite of microvascular and macrovascular endpoints and the secondary outcomes were microvascular and macrovascular morbidity and mortality separately. Although, metformin was not associated with an improvement in the combined primary outcome, it was associated with an improvement in the secondary macrovascular outcomes (HR 0.61, 95% CI 0.40, 0.94; *p* = 0.02).

#### 2.2.3. Increased Mortality in Combination with SU

Another criticism is based around the increased mortality seen in those treated with combination of SU and metformin in UKPDS. In the UKPDS, early addition of metformin in SU-treated patients was associated with an increased risk of diabetes-related death (96%, 95% CI 2, 275, *p* = 0.039) compared with continued SU alone, although a sub-group analysis did not show such association with a combination of SU and metformin [2]. A meta-analysis looking at the safety of combination of SU and metformin showed an increased risk for a composite end-point of CVD hospitalisations and mortality but no significant effects of this combination therapy was seen on either CVD mortality or all-cause mortality alone [29]. An observational cohort study also showed a significantly increased mortality in individuals treated with combination of various SUs (glibenclamide and chlorpropamide) and biguanides compared to other combinations and monotherapies [30]. However, it was acknowledged that patients who were on combined treatment were more likely to be obese and showed more severe associated metabolic abnormalities. Most of the individuals on combination therapy were treated with older longer-acting SUs like glibenclamide. Newer 3rd generation SUs like glimepiride, which have lower affinity for myocardial ATP-dependent potassium channels compared to glibenclamide [31], could show a lower detrimental impact when used with biguanides [30] and animal models have confirmed that compared to conventional SUs, glimepiride has less harmful cardiovascular effects [32]. The newer generation of SUs also appear to confer less risk of hypoglycaemia compared to older generation of SUs used in UKPDS [33]. As a result, these shorter-acting SUs could be a safer option when combined with metformin compared to longer-acting SUs.

#### 2.2.4. Impact of Other Interventions

Critics also raised concerns because of the impact of other interventions including optimisation of blood pressure control and lipid profile during the long follow-up period in UKPDS, which could have affected the outcomes. The Steno-2 study demonstrated that early multifactorial risk reduction does lead to improvement in outcomes including CVD death [34] and treatment intensification is required over the course of T2D timeline.

#### 2.2.5. UKPDS vs Newer Cardiovascular Outcome Trials (CVOTs)

It is important to remember that UKPDS was first reported more than two decades ago looking at effects of intensive GLT on long-term outcomes and was not designed to test the efficacy of individual glucose-lowering agents. Similarly, Action to Control Cardiovascular Risk in Diabetes (ACCORD) [35], Action in Diabetes and Vascular Disease: Preterax and Diamicron Modified Release Controlled Evaluation (ADVANCE) [36] and Veterans Affairs Diabetes Trial (VADT) [37] were trials comparing the effects of intensive vs standard glucose control on cardiovascular events in T2D populations with established or high risk of cardiovascular disease and were not drug efficacy trials per se. In all these three trials, individuals in both intensive and control arms received metformin therapy. In ACCORD, 94.7% individuals were treated with metformin in the intensive therapy arm compared to 86.9% in the control arm [35], in ADVANCE, 74% of participants received metformin in the intensive control group vs 67% in the standard control group [36] and finally in VADT [37], in both study groups, individuals either received metformin or glimepiride based on BMI in combination with rosiglitazone. As a result, it becomes difficult to draw any direct inferences regarding metformin efficacy based on described trial designs. Consequently, changes in trial standards over these years complicates drawing direct comparisons between metformin and newer agents. The history of CVOTs can broadly be categorised into pre-2008 and post-2008 periods after the US Food and Drug Administration (FDA) introduced the new guidelines for cardiovascular evaluation of GLTs largely focussed around the composite end-point of Major Adverse Cardiovascular Events (MACE) following the discovery of increased adverse cardiovascular events associated with rosiglitazone use [38,39]. Hence, considerable caution is required when comparing older and newer trials, which are more standardised in design and amenable to meta-analysis [26]. It is also worth noting that the modern CVOTs are relatively short trials recruiting mostly high-risk individuals who are unrepresentative of the general population. Hence, there is a need to widen the scope of these CVOTs by facilitating more pragmatic designs employing diverse recruitment strategies with flexibility in design, ensuring extended follow-up and exploring the additive effects of different combinations of GLTs [40].

#### 2.2.6. The European Society of Cardiology (ESC) 2019 Guidelines on Diabetes, Pre-diabetes, and CVD

The MACE outcome trials of “newer” GLTs namely the glucagon-like peptide-1 receptor agonists (GLP-1RA) and sodium-glucose co-transporter 2 inhibitors (SGLT2i) have demonstrated cardiovascular benefits in large CVOTs. Based on these trials, the American Diabetes Association (ADA) and the European Association for the Study of Diabetes (EASD) recommend early addition of these agents to baseline metformin therapy in individuals with established cardiorenal disease or high risk for cardiorenal disease [41]. The ADA/EASD recommendations retain metformin as the fundamental starting therapy in individuals with new diagnosis of uncomplicated T2D.

However, In 2019, the ESC guidelines on diabetes, pre-diabetes, and CVD recommended use of either SGLT2i or GLP-1RA in individuals with established ASCVD or high/very high cardiovascular risk as first-line, whether they are treatment-naïve or already on metformin [42]. Similarly, others have also proposed an alternate algorithm for individuals with T2D and ASCVD in which SGLT2i and GLP-1RA are the first line irrespective of HbA1c targets [43]. It is important to remember that metformin was the baseline therapy in most participants in these CVOTs and the cardiovascular benefits of SGLT2i and GLP-1RA remain largely unknown in treatment-naïve individuals. The current advice from ESC may be a step too far to start these newer agents in treatment-naïve individuals before metformin.

It may appear as if there is an area of difference between ADA/EASD recommendations and ESC guidelines with regards to positioning of metformin. However, on closer inspection there are more similarities between the two as ADA/EASD emphasizes that patients at high risk for cardiorenal disease should be treated with SGLT2i or GLP-1RA independent of HbA1c. In addition, most patients with T2DM progress to requiring combination therapy soon after baseline metformin therapy. A recent meta-analysis examining comparative effectiveness of GLTs concluded that the use of metformin as first-line treatment in treatment-naïve individuals with low cardiovascular risk remains justified and given the lack of evidence, they could not reach a conclusion about the optimal initial treatment of treatment-naïve patients at increased cardiovascular risk [44]. Future studies will need to address whether SGLT2i and GLP-1RAs should be used for the prophylaxis of ASCVD and CKD in uncomplicated newly diagnosed treatment-naïve individuals with T2D to address this further.

### 2.3. What Does Post-UKPDS Cardiovascular Data Tell Us about Metformin; Is It Good or Not So Good?

In this section, we look at the major post-UKPDS meta-analysis and systematic reviews exploring the all-cause mortality and cardiovascular outcomes of metformin versus placebo, no therapy, or active comparators to explore whether these also consolidate UKPDS findings. We have divided these into those not supporting UKPDS findings and those supporting UKPDS findings, at least partially if not fully.

#### 2.3.1. Meta-analysis Not Supporting UKPDS Findings

A meta-analysis by Griffin et al., examined randomised trials looking at the impact of metformin on CVD exclusively compared to diet, lifestyle or placebo [45]. All outcomes, with the exception of stroke, favoured metformin, but none achieved statistical significance and they concluded that there is uncertainty about whether metformin reduces risk of CVD among individuals with T2D. However, they acknowledged that this was mainly due to lack of evidence from a randomised-controlled CVOT comparing metformin against placebo. Similarly, Boussageon et al., did not find any evidence supporting metformin’s beneficial impact on all-cause mortality, cardiovascular mortality or any major macrovascular complications in their meta-analysis of randomised-controlled trials (RCTs) evaluating metformin cardiovascular efficacy compared to placebo, diet, no treatment as well as studies looking at metformin as an add-on therapy and studies of metformin withdrawal [46]. They suggested a need for further studies to clarify this as the existing number and qualities of studies were insufficient.

#### 2.3.2. Meta-analysis/Systematic Reviews (Partially or Fully) Supporting UKPDS Findings

In a large meta-analysis by Han et al. looking at cardiovascular mortality between metformin and non-metformin groups, the adjusted HR was 0.81 (95% CI 0.79, 0.84; *p* < 0.00001) in favour of metformin [47]. Similarly, the adjusted HR between the metformin and non-metformin groups for all-cause mortality was 0.67 (95% CI 0.60, 0.75; *p* < 0.00001). Similarly, in a previous meta-analysis a significant cardiovascular benefit was observed in metformin versus placebo/no therapy trials with odds ratio (OR) of 0.79 (95% CI 0.64, 0.98; *p* < 0.031), but this superiority was not seen with metformin in active-comparator trials with OR of 1.03 (95% CI 0.72, 1.77; *p* = 0.89) [48]. They proposed that the reason behind this could be that metformin cardiovascular benefits are dependent on its glucose-lowering properties hence the observed superiority only compared to placebo or no therapy groups. However, the UKPDS data showed improvements in cardiovascular outcomes with metformin compared to insulin or SU despite similar glycaemic improvements. Campbell et al., performed an extensive meta-analysis comparing several cardiovascular outcomes of metformin against non-metformin therapy [49]. This supported UKPDS findings to an extent showing that all-cause mortality improved with metformin therapy compared to active comparators, but this was not seen in trials comparing metformin against diet-controlled diabetes. They thought that the difference could be due to diet therapy being a more likely option for individuals with early or less severe disease. A 2008 systematic review exploring cardiovascular outcomes of oral GLTs showed that metformin therapy was associated with a decreased risk of cardiovascular mortality compared with both placebo and any other oral GLT [50]. However, it failed to establish metformin’s superiority on all-cause mortality or cardiovascular morbidity suggesting that compared to other oral GLTs and placebo, metformin overall appeared only moderately protective. Finally, Crowley et al., looked at the cardiovascular outcomes of metformin in high-risk populations with CHF, chronic kidney disease (CKD) and chronic liver disease (CLD) [51]. They showed improvement in key clinical outcomes including all-cause mortality and MACE with metformin use even in individuals with moderate CKD, CHF, or CLD compared to non-metformin regimens, proving that metformin is not only safe but also potentially beneficial in such high-risk populations.

Table 1 below provides a summary of these systematic reviews and meta-analyses with main outcome results and important conclusions.

In summary, the results of these reviews are inconclusive in respect to the cardiovascular impact of metformin. It was acknowledged that a lot of this uncertainty was due to lack of a large randomised double-blind, placebo controlled trial with dedicated MACE end-points [45], lack of long-term evaluations [26] and the unexplained deleterious effect of the combination of metformin plus SU from the UKPDS data [25,46]. Additionally, differences in patient characteristics, study designs and duration could provide an explanation for the discrepancies observed.

In the majority of these meta-analyses and systematic reviews the balance does tilt in favour of metformin. Given its beneficial effects on HbA1c, weight and cardiovascular mortality and relative safety profile compared to most of the other GLTs, it is reasonable to support metformin as first-line therapy for individuals with new diagnosis of uncomplicated T2D [52].

## 3. Additional Benefits of Metformin Therapy

### 3.1. What Are the Additional Benefits of Metformin Use Compared to Other GLTs Besides the Pleiotropic Cardiovascular Effects?

The ADA/EASD published an update to the management of hyperglycaemia in individuals in with T2D diabetes which recommends use of GLP-1RA and SGLT2i as a second line agent not only in those with established ASCVD but also in those without ASCVD but at high risk of ASCVD [53]. Metformin remains first-line after lifestyle interventions (diet and exercise) due to key safety and efficacy reasons. Some of these are discussed below.

#### 3.1.1. Goals of Management in T2D

CVD is the most common co-morbidity in individuals with T2D and consequently CVOTs tend to use mostly high-risk populations. However, for newly diagnosed individuals managed mostly in primary care the main goal of management of T2D is optimisation of glycaemic control using HbA1c. This can be addressed using metformin as a first-line therapy.

It is crucial to escalate therapy after metformin as within 2-years of initiating metformin monotherapy, over 30% of patients require an additional agent and within 3-years over 50% require combination therapy [54]. ADA/EASD emphasis the need for early addition of SGLT2I or GLP-1RA for individuals with high risk for cardiorenal disease and to consider initial combination therapy for those with sub-optimal glycaemic control based on outcomes of VERIFY trial [53,55].

#### 3.1.2. Safety and Tolerability in Elderly Population

The age range for MACE outcome trials of GLP-1RA and SGLT2i have been around 60 to 65 years [56] and does not include frail individuals who form a large cohort of T2D. For such individuals, metformin with convenient dosing schedule, low risk of hypoglycaemia and good tolerability requiring little monitoring appears to be a safe and effective option [57].

#### 3.1.3. Efficacy of Metformin as a Glucose-lowering Agent

Metformin is an effective agent in lowering HbA1c (1.0–1.5%) [58]. HbA1c not only reflects longer-term glycaemic control but improvement in HbA1c correlates with reduced risk of cardiovascular complications [59,60,61]. In a recent meta-analysis, metformin reduced mean HbA1c by −0.92% (95% CI −1.07, −0.77) in treatment-naïve individuals which was lower only to canagliflozin (−1.02% [95% CI −1.68, −0.36]) and oral semaglutide (−1.10%, 95% CI −1.7, −0.45) amongst oral GLTs [44]. Metformin has been found to improve glycaemic variables significantly (both fasting blood glucose and HbA1c) compared to placebo in a dose-dependent fashion at dosages of 500 to 2000 mg daily, with maximal benefits observed at upper limits of recommended daily dosage [62]. All dosages were well-tolerated with no increase in hypoglycaemia. In a comparative analysis of effectiveness and safety of oral medications for T2D, metformin was found to be either more effective or equally potent to other available agents with lower risk of serious complications [63].

#### 3.1.4. Side Effect Profile of Metformin Including High Risk Groups

The main side effect of metformin is gastrointestinal (GI), which can be negotiated easily by gradual dose-titration, taking the medication with food or switching to a slow-release preparation [64]. The incidence of lactic acidosis sometimes associated with metformin use is extremely rare and is generally indistinguishable from the background rate in the overall population with diabetes [65]. In 77,601 individuals treated with metformin for T2D, only 35 events of lactic acidosis were recorded of which none were fatal, and no significant difference was observed among individuals with normal or impaired kidney function [66]. In fact, the incidence in those taking metformin is similar to those taking other GLT and it can be safely commenced and continued in those with eGFR >45mL/min/1.73 m^2^ without requiring any regular monitoring or surveillance [67]. In the COSMIC study, the incidence of serious adverse events was similar between metformin and other treatment groups and no case of lactic acidosis was reported in the metformin group [68]. Rachmani et al. reported no cases of lactic acidosis in individuals with CHF (New York Heart Association (NYHA) III-IV), chronic obstructive pulmonary disease (COPD) and CKD (serum creatinine 130–220 μmol/L) [69], which are regarded as traditional contraindications to use of metformin due to potentially increased risk of lactic acidosis. They concluded that metformin can be continued safely in those with mild renal impairment and in those with CHF and COPD. In this study, discontinuation of metformin in these high-risk individuals was associated with a rise in HbA1c, weight gain and modest increase in low-density lipoprotein (LDL) cholesterol compared to those who continued the medication [69]. In a review of available literature, Tahrani et al., summarised that metformin use in individuals with HF might be associated with lower mortality and morbidity, with no increase in hospital admissions and no documented increased risk of lactic acidosis [70]. They concluded that the decision to stop or continue metformin in the presence of HF should be individualised to the patient until further evidence is available. Overall, it appears that mortality in patients receiving metformin who even develop lactic acidosis is linked to underlying disease rather than to metformin being a toxic medication [71].

#### 3.1.5. Impact on Weight

Metformin is considered weight-neutral but is also known to cause weight loss [72] with mean value of 1.1 kg vs placebo [73]. This is in contrast to SU, pioglitazone and insulin, which all induce weight gain. In a systematic review and meta-analysis, metformin decreased body weight more than DPP-4 inhibitors (−1.3kg, 95% CI −1.6, −1.0) [52]. Weight loss is independently associated with improvement in cardiometabolic risk factors [74]. The 1.7 kg weight loss with metformin versus a 0.3 kg gain with placebo alone explained 64% of the beneficial metformin effects on diabetes risk in the Diabetes Prevention Program (DPP) [75]. In the HOME trial, combining metformin with insulin was associated with improved glycaemic control (mean HbA1c 51.9 [6.9] vs 59.6 mmol/mol [7.6%]; *p* < 0.0001), reduced insulin requirements (63.8 vs 71.3 IU; *p* < 0.0001), reduced weight gain (−0.4 vs +1.2 kg; *p* < 0.01) and decreased plasma LDL cholesterol (−0.21 vs −0.02 mmol/L; *p* < 0.01) compared to placebo [76].

#### 3.1.6. Prevention of Diabetes

One of the goals of therapies in T2D management is to prevent the development of the disease in the first place and metformin was found to lower the incidence of diabetes by 31% compared to placebo after 2.8 years in the DPP [77], making it an excellent choice in individuals with high risk of developing diabetes. The impact was greater in those who were more obese, had a higher fasting glucose or a history of gestational diabetes. The Diabetes Prevention Program Outcomes Study (DPPOS) looked at the long-term effects of metformin and showed a RR of 18% over 10 and 15 years post-randomisation including reduction in adverse cardiovascular outcomes with metformin use [77]. Metformin has the strongest evidence-based and long-term safety profile as the pharmacologic therapy of choice for diabetes prevention [78].

#### 3.1.7. Low Risk of Hypoglycaemia

Metformin is not associated with increased risk of hypoglycaemia unless used in combination with insulin or insulin secretogogues like SU and hence does not require self-monitoring of blood glucose when used as a monotherapy. This feature makes it an attractive first choice for newly diagnosed individuals with T2D in addition to its wider global availability and cost-effectiveness. Combining metformin with insulin also reduces risk of hypoglycaemia with less weight gain [79,80].

## 4. Key Questions on the Current Role of Metformin

### 4.1. Should Metformin be the First-Line Treatment in T2D?

In order to answer this question, let us first define an ideal glucose-lowering agent. An ideal glucose-lowering agent should not only be safe and effective but should also be durable, should be suitable for use at all stages of diabetes, across a range of individuals with range of co-morbidities, should be simple to administer with good adherence and suitable for use in combination with other agents [81]. We are not there yet, but a single agent, which fills most of these criteria above, is metformin and before definitely replacing metformin with another GLT as first-line agent, we need to be sure of the following.

#### 4.1.1. Is There is a Clear Evidence in Head-to-head Trials of a Benefit of Other Agents Over Metformin, Either in Cardiovascular Benefit or Cost-Effectiveness?

##### SGLT2i and GLP-1RA

So far, we do not have any direct head-to-head primary CVOTs comparing metformin against either SGLT2i or GLP-1RA, but cost will remain a consideration even if either SGLT2i or GLP-1RA were found to be superior. In addition, there are significant intra-class differences in terms of the cardiovascular outcomes within both SGLT2i and GLP-1RA, and not all agents are equally potent or effective.

##### SU

A multicentre randomized, double-blind, placebo controlled trial of metformin and glipizide on cardiovascular outcomes in individuals with T2D and coronary artery disease (CAD) found that treatment with metformin for 3 years substantially reduced MACE compared with glipizide (HR of 0.54, 95% CI 0.30, 0.90; *p* = 0.026) [82]. In an observational study of 10,920 individuals with T2D and concomitant HF, treatment with metformin monotherapy was associated with a low risk of mortality compared with either SU or insulin monotherapy [83].

##### Dipeptidyl Peptidase-4 (DDP4) Inhibitors

For DDP4 inhibitors, the MACE outcomes have been neutral in terms of the cardiovascular benefits [84,85,86,87]. The use of saxagliptin was associated with higher risk of HF compared to placebo (HR 1.27, 95% CI 1.07, 1.51; *p* = 0.007) [85], albeit not a primary outcome of the study. Adjusted HR for a composite of CVD events including hospitalizations for ischemic stroke, MI and HF, and hypoglycaemia were all found to be statistically lower for metformin compared to DPP4 inhibitors as a class (0.87, 95% CI 0.79, 0.94) in a longitudinal study of 123,050 individuals with T2D followed over several years [88]. Even in major CVOTs of DPP4 inhibitors, baseline metformin use was associated with a trend towards improved cardiovascular outcomes (HR 0.92, 95% CI 0.84, 1.01) compared to baseline metformin nonusers (HR 1.10, 95% CI 0.97, 1.26) suggesting that baseline metformin status may have a moderating effect on CVOTs of DPP4 inhibitors [89]. However, it is difficult to establish a causal relationship here without incorporating statistical adjustment for other differences like rates of CHF or CKD etc.

##### Thiazolidinediones (TZDs)

In terms of TZDs, rosiglitazone was withdrawn by the FDA after it was found to be associated with adverse cardiovascular outcomes, especially HF hospitalisations and death [39,90]. A RCT looked at efficacy and safety of pioglitazone versus metformin to provide evidence in favour of pioglitazone over metformin as an alternate first-line therapy [91]. Although some parameters like fasting blood glucose, triglyceride (TG), high-density lipoprotein (HDL) and urine albumin to creatinine ratio improved in favour of pioglitazone, others improved in favour of metformin including total cholesterol, LDL and body weight with similar HbA1c reduction in both groups [91]. The study did not examine the MACE outcomes directly. In addition, the side effect profile of pioglitazone including risk of fluid retention and HF makes it difficult to choose pioglitazone as a first-line agent over metformin [92].

#### 4.1.2. Is There Evidence That Metformin is Harmful or Mitigated the Beneficial Effects of Cardio-Protective GLT Such as SGLT2i or GLP-1RA When Used in Combination?

There has been an immense proliferation in the evidence suggesting the beneficial cardio-protective effects of SGLT2i and GLP-1RA especially in the last 5 years. Despite all this, the uptake of these medications outside of diabetologists domain remains low [93,94]. It is crucial to avoid therapeutic inertia [95] and therapy beyond metformin is escalated in a timely manner.

As a result of its unique mechanism of action and lack of hypoglycaemia, metformin pairs well with all other GLTs and is the most common agent used in single-tablet fixed-dose combinations [96]. For these reasons, metformin was the baseline therapy in the majority of recent MACE outcome trials assessing the newer agents including GLP-1RA and SGLT2i therapies [43]. Hence, the cardiovascular benefits for SGLT2i and GLP-1RA therapy are seen mostly in individuals who were already taking metformin and we have limited information about the precise interaction of these relatively “newer” agents with metformin [97], which are unlikely to be detrimental.

##### Metformin and SGLT2i

SGLT2i have been found to have favourable influence on HF [98,99,100]. As mentioned, the majority of individuals included in these trials were on background metformin therapy but there are some concerns that metformin may attenuate the magnitude of HF benefits of SGLT2i [101]. In one large trial, empagliflozin reduced the risk of cardiovascular death more without metformin (HR 0.46, 95% CI 0.32, 0.68) than with metformin (HR 0.71, 95% CI 0.54, 0.94) although this interaction did not reach statistical significance (*p* = 0.07) [102]. Similarly, in another large scale trial looking at the impact of canagliflozin, the beneficial effects of canagliflozin therapy on cardiovascular death or HF were greater in those who were not on metformin (HR 0.64, 95% CI 0.50, 0.82) compared to those who were on metformin baseline therapy (HR 0.88, 95% CI 0.72, 1.08; *p* = 0.03) [103]. This raises further concerns about the impact of metformin pre-treatment on HF benefits realised with SGLT2i therapy. However, caution is required when interpreting these results and drawing conclusions. These CVOTs were primarily conducted to investigate the safety outcomes of SGLT2i therapy as mandated by FDA. All these analyses exploring possible interaction of metformin with SGLT2i therapy should be viewed only as hypothesis generating in view of lack of statistical power and multiple testing adjustment, bearing in mind heterogeneity of treatment effect with metformin in these trials. To clarify whether metformin attenuates the HF effects of SGLT2i, then the analysis of these trials must account for factors that could be casually related to the use of metformin and outcomes.

Although we cannot draw exact conclusions about the effect of metformin and SGLT2i interaction on HF outcomes, individually both these medications act by mechanisms that are believed to ameliorate cardiomyopathy [104,105,106]. Metformin works by activating AMP-activated protein kinase (AMPK) [107] and there is evidence that SGLT2i in addition to stimulating sirtuin 1 (SIRT1) [108] also activates AMPK [105], and thus it is possible that any direct cardioprotective effects of SGLT2i that depend on AMPK activation could be attenuated [101]. However, these hypotheses need further exploration.

It is worth remembering that studies have shown that combinations of metformin with SGLT2i is not only well-tolerated but effective in improving glycaemic control and promoting weight loss [109,110,111]. As mentioned already, metformin was the baseline therapy in majority of the individuals recruited to CVOTs of SGLT2i therapy [112]. In treatment-naïve individuals, early combination therapy with metformin and canagliflozin led to a dose-dependent decrease in HbA1c and weight [109] and can be considered as an option in appropriate individuals where weight is a major concern.

##### Metformin and DPP4 Inhibitors

Meta-analysis of the three major CVOTs examining DPP4 inhibitors [84,85,86], showed that baseline metformin users experienced a trend towards improved cardiovascular outcomes when combined with DPP4 inhibitors (HR 0.92, 95% CI 0.84, 1.01) compared to baseline metformin non-users who in fact showed a trend towards harm (HR1.10, 95% CI 0.97, 1.26) [89]. The underlying mechanisms presumed were the possible increase in glucagon-like peptide-1 (GLP-1) levels by metformin [113] plus direct inhibition of the enzyme dipeptidyl peptidase-4 (DPP4) by the DPP4 inhibitors, thus acting synergistically [89]. Another possibility could be an increase in endothelial progenitor cells (EPC) function and a decline in pro-inflammatory cytokines expression [114]. In a post-hoc analysis of SAVOR-TIMI 53, although metformin use was associated with no difference in risk for the composite end-point (HR 0.92, 95% CI 0.76, 1.11), there was a lower risk of all-cause mortality with metformin use (HR 0.75, 95% CI 0.59, 0.95) [115].

##### Metformin and GLP-1RA

Compared to a combination of metformin plus SU, metformin plus GLP-1RA was associated with RR of 0.77 (95% CI 0.51, 1.17) for total mortality, 0.89 (95% CI 0.47, 1.68) for cardiovascular mortality, and 0.82 (95% CI 0.55, 1.21) for the combined end-point in a study of Danish population without prior MI or stroke [116]. In the Harmony outcomes trial, those on baseline metformin therapy did slightly better (HR 0.77, 95% CI 0.65–09.2) than those who were not on metformin (HR 0.79, 95% CI 0.62, 1.00) [117]. A review of 21 studies looking at combination of metformin with incretin-based therapies (DPP4 inhibitors and GLP-1RA), showed that combination of metformin with long-acting GLP-1RA caused a more pronounced reduction in HbA1c than with short-acting exenatide and DPP4 inhibitors (both *p* < 0.001) [118]. However, both combinations (metformin/GLP-1RA and metformin/DPP4 inhibitors) were associated with low risk of adverse events, including hypoglycaemia proving these combinations are safe and effective.

#### 4.1.3. If Metformin Use Significantly Delayed the Addition of Other Agents?

Metformin pairs well with most oral and injectable GLTs and there is no evidence that metformin leads to delay in escalation of therapy. The ADA/EASD recommends early addition of agents with proven cardiovascular benefits where indicated, irrespective of baseline or individualised HbA1c [41].

#### 4.1.4. If Metformin Was More Harmful or Expensive Compared to Other GLT?

Metformin is generally considered to be a safe option as a GLT. In a systematic review of observational studies involving 34,000 patients looking at comparative safety and effectiveness of metformin, its use was associated with reduced mortality compared with controls (mostly SU therapy): 23% vs 37% (pooled adjusted risk estimate 0.80, 95% CI 0.74, 0.87; *p* < 0.001) [119].

##### Metformin Use in HF and CKD

As mentioned above, HF and CKD were traditionally considered as conditions associated with increased risk of lactic acidosis in metformin users. In this section, we look at some key data around use of metformin in individuals with HF and CKD individually.

(i) Metformin and HF

In a UK study of primary care data of individuals with new diagnosis of HF, compared with patients who were not exposed to GLTs, the use of metformin monotherapy (adjusted OR 0.65, 95% CI 0.48, 0.87) or metformin with or without other agents (adjusted OR 0.72, 95% CI 0.59, 0.90) was associated with lower mortality [120]. Even in individuals with advanced HF (NYHA III and IV and left ventricular ejection fraction (LVEF) 24 ± 7%), one-year survival rate in metformin-treated (91%) individuals was greater than those individuals who were not on metformin (76%) with RR of 0.37 (95% CI 0.18, 0.76; *p* = 0.007) [121]. However, after multivariate adjustment for demographics, cardiac function, renal function, and HF medications, metformin therapy was associated with only non-significant trend for improved survival suggesting need for further prospective studies in this regard.

Compared to SU monotherapy, use of metformin monotherapy in new users of oral GLTs with incident HF was associated with fewer deaths (33% vs 52%) with HR of 0.70 (95% CI 0.54, 0.91) [122]. Metformin therapy was found to be associated with lower rate of readmission for HF (HR 0.92, 95% CI 0.92, 0.99) in a retrospective cohort of individuals with diabetes discharged from hospital with the principal diagnosis of HF while there was a higher risk of readmission for HF with TZD treatment (HR 1.06, 95% CI 1.00, 1.09) [123]. In the same study, metformin use was associated with lower mortality when compared to treatment with either insulin or SU (24.7% vs 36%, *p* < 0.0001) [123].

In a study comprising of 16,691 patients with diabetes and established atherothrombosis, the mortality rates were 6.3% (95% CI 5.2, 7.4) with metformin and 9.8% (95% CI 8.4, 11.2) without metformin; the adjusted HR was 0.76 (95% CI 0.65, 0.89; *p* < 0.001) [124]. Association with lower mortality was noticeable in patients with a history of CHF, patients older than 65 years and patients with an eGFR of 30 to 60 mL/min/1.73m^2^. The results suggest a possible role of metformin for secondary prevention of CVD compared to some of the older agents.

Overall, these findings establish a safe profile of metformin in individuals with HF and in 2006, FDA removed CHF as a contraindication for metformin use [125]. A recent study also established the lower risk of HF hospitalisation with metformin use compared to individuals who have never been treated with metformin (HR 0.57, 95% CI 0.53, 0.62) [126]. In a 2018 position statement, the ESC states that metformin might be safe and efficacious in individuals with T2D and HF based on the results of large observational studies and concern that metformin may cause metabolic acidosis is not justified [127]. They recommended metformin as first-line treatment for individuals with T2D and HF who have preserved or moderately reduced renal function (eGFR > 30 mL/min).

(ii) Metformin and CKD

The use of metformin has been found to be safe in stable CKD. In a large trial comparing metformin users vs non-users with diabetes and CKD, metformin use was associated with reduced risk of all-cause mortality (HR 0.49, 95% CI 0.36, 0.69), cardiovascular death (HR 0.49, 95% CI 0.32, 0.74), the cardiovascular composite (HR 0.67, 95% CI 0.51, 0.88) and the kidney disease composite (HR 0.77, 95% CI 0.61, 0.98) [128]. Associations with end-stage renal disease (ESRD) (HR 1.01, 95% CI 0.65, 1.55] were not significant. The study concluded that metformin may be safe for use in CKD and may lower the risk of death and cardiovascular events in individuals with stage 3 CKD. In the UK, National Institute for Health and Care Excellence (NICE) recommends to review the dose of metformin only if eGFR is below 45 mL/min/1.73 m^2^ and only stopping metformin if the eGFR is below 30 mL/min/1.73 m^2^ [129].

##### How Does Side Effect Profile of Metformin Compare to Other Oral GLTs?

The newer GLTs namely DPP4 inhibitors, SGLT2i and GLP-1RA each have specific side effect profile as individual categories, some of which though rare can be potentially serious and are not seen with metformin use.

(i) DPP4 Inhibitors

DPP4 inhibitors have been known to cause side effects like skin reactions, angioedema, nasopharyngitis and rarely pancreatitis, which we do not see with metformin. There have been concerns raised about increased risk of infections associated with use of DPP4 inhibitors due to its immune modulating effects. In a meta-analysis of safety and efficacy of incretin-based therapies, DPP4 inhibitor use was associated with an increased risk of infection with RR 1.2 (95% CI 1.0, 1.4) for nasopharyngitis and 1.5 (95% CI 1.0, 2.2) for urinary tract infection (UTI) compared to placebo or active comparators [130]. A nested case-control study of the WHO database Vigibase also found increased reporting of infections for individuals using DPP4 inhibitors compared with users of biguanides (OR 2.3 (95% CI 1.9, 2.7) [131]. Although a recent meta-analysis of RCTs did not find an increased risk of infections with DPP4 inhibitors, there was a non-significant trend of increased infection rate with DPP4 inhibitor use compared to metformin (OR 1.22 (95% CI 0.95, 1.56; *p* = 0.12) [132].

(ii) SGLT2i

SGLT2i use has been associated with increased risk of urinary and genital tract infections. The increased risk of UTIs suggested by some of the data however was not proved in meta-analyses of large trials. A meta-analysis looking at effects of SGLT2i on UTIs and genital infections did not demonstrate increased risk of UTIs compared to control (RR 1.05, 95% CI 0.98 to 1.12) but did suggest increased risk of genital infections with SGLT2i (RR 3.30, 95% CI 2.74 to 3.99) [133]. Similarly, another meta-analysis on risk of infections with SGLT2i use also confirmed increased risk of genital tract infections compared to placebo (RR 3.37, 95% CI 2.89, 3.93) or active comparator (RR 3.89, 95% CI 3.14, 4.82) but not UTI vs placebo (RR 1.03, 95% CI 0.96, 1.11) or active comparator (RR 1.08, 95% CI 0.93, 1.25) [134]. A large US population-based study also failed to establish this increased risk of UTI with SGLT2i therapy compared to other GLTs [135]. It is likely that T2D as a condition is associated with increased risk of UTI rather than the SGLT2i itself [136].

A serious adverse event associated with SGLT2i use is euglycaemic ketoacidosis, but the risk is negligible especially if the drug is properly prescribed [137]. Similarly, Fournier’s gangrene, which is a life-threatening necrotizing fasciitis of the perineum, has been associated with SGLT2i but again the frequency of such events is not precisely known but presumed to be very small [138]. Use of canagliflozin in the CANVAS trial was associated with an increased risk of lower limb amputations [99], however, this was not shown in the subsequent CREDENCE trial [139].

(iii) GLP-1RA

The GLP-1RA use as a class is associated with primarily GI side effects like nausea, vomiting and diarrhoea and rarely pancreatitis. Metformin can also cause GI side effects. Approximately 25% of individuals experience GI side effects as a result of metformin therapy [140] particularly at the start of therapy and 5% of those discontinue metformin due to these side effects [141]. In contrast, results from a UK audit database showed that approximately 12.1% of patients reported GI side effects to liraglutide therapy but the total number of individuals who discontinued liraglutide within 26 weeks of treatment including those who discontinued due to GI side effects was higher at 13.6% [142]. However, these GI side effects seem to be more pronounced with shorter acting formulations compared to longer acting formulations [143]. Like DPP4 inhibitors, which also target incretin-pathway to lower blood glucose, GLP-1RA use is also associated with increased risk of infections, skin reactions including angioedema and rarely pancreatitis [144]. There are also concerns about the association of chronic GLP1-RA therapy with development of thyroid cancer especially in animal models [145].

##### Cost of Metformin Therapy Compared to Other Commonly Prescribed GLTs

Metformin is one of the most cost-effective agents in the modern day management of T2D as shown by the Table 2 below which shows the average one-month cost of metformin therapy compared to some of the other commonly prescribed agents in UK. This is an important consideration as 79% of adults with diabetes live in low- and middle-income countries [146].

## 5. Conclusions

Metformin possesses many of the features expected of an ideal GLT; it is safe, effective, cheap, pairs well with most other agents with little to no adverse interaction and is available globally [147]. It is unlikely that dedicated prospective trials looking at MACE outcomes to fully elucidate the cardiovascular benefits realised in experimental models and observational data will ever happen and metformin’s current role as a “foundation therapy” for newly diagnosed individuals with T2D may continue to be both justified and equally challenged. This is especially true when there is an increasing emphasis to transition existing algorithms from an approach based solely on HbA1c to a more comprehensive strategy targeting additional patient factors especially CVD prevention. Until further safety data becomes available for SGLT2i and GLP-1RA use in treatment-naïve individuals, we recommend that not only the efficacy but also the cost and the long-term safety profile should guide decisions in clinical practice and metformin should continue to be used as a first-line therapy for newly diagnosed individuals with T2D. The key message is to avoid therapeutic inertia, as the uptake of these “newer” GLTs with proven cardiovascular benefits remains generally low and to consider early addition of these agents to baseline metformin therapy where indicated.

## Figures and Tables

**Table 1:** Cardiovascular meta-analysis and systematic review data on metformin.

Author/Source/Year/ Reference	Number of Studies	Primary Aim	Comparator/s	HR/OR (95% CI) (Metformin vs Comparators)	Conclusion(s)
Griffin et al./*Diabetologia*/2017 [45]	13 (RCTs only)	Impact of metformin on CVD	Diet, lifestyle or placebo	All-cause mortality: 0.96 (0.84, 1.09) Cardiovascular mortality: 0.97 (0.80, 1.16) MI: 0.89 (0.75, 1.06) Stroke: 1.04 (0.73, 1.48)	All outcomes, except stroke, favoured metformin but none achieved statistical significance
Bossageon et al./*PLoS Med*./2012 [46]	13 (RCTs only)	Cardiovascular efficacy of metformin	Diet, placebo, no treatment; metformin as an add-on therapy; and metformin withdrawal	All-cause mortality: 0.99 (0.75, 1.31) Cardiovascular mortality: 1.05 (0.67, 1.64) MI: 0.90 (0.74, 1.09) Stroke: 0.76 (0.51, 1.14) CHF: 1.03 (0.67, 1.59)	Could not exclude whether metformin use increases or decreases the risk of all-cause mortality or cardiovascular mortality.
Han et al./*Cardiovasc Diabetol*/2019 [47]	40	Effect of metformin in individuals with CAD	No metformin controls	All-cause mortality: 0.67 (0.60, 0.75) Cardiovascular mortality: 0.81 (0.79, 0.84) All-cause mortality with MI at baseline: 0.79 (0.68, 0.92) All-cause mortality with HF at baseline: 0.84 (0.84, 0.87)	Metformin reduced cardiovascular mortality, all-cause mortality and CV events in CAD patients.
Lamanna et al./*Diabetes Obes Metab.*/2011 [48]	35 (RCTs only)	Effect of metformin on cardiovascular events and mortality	Placebo/no therapy/active comparators	All-cause mortality: 1.10 (0.80, 1.51) Cardiovascular events: 0.94 (0.82, 1.07) vs placebo/no therapy: 0.79 (0.64, 0.98) vs active comparator: 1.03 (0.72, 1.77)	Cardiovascular benefit of metformin was only demonstrated vs placebo/no therapy but not in active-comparator trials.
Campbell et al./*Ageing Res Rev.*/2017 [49]	53	Effect of metformin on all-cause mortality	Non-metformin therapies (Any controls not receiving metformin)	All-cause mortality: vs non-diabetics: 0.93 (0.88, 0.99) vs non–metformin diabetic controls: 0.72 (0.65, 0.80) vs diet-controlled diabetics: 0.91 (0.68, 1.22) vs insulin users: 0.68 (0.63, 0.75) vs SU users: 0.80 (0.66, 0.97) CVD (Diabetics only): vs non–metformin control: 0.76 (0.66, 0.87) vs diet-controlled diabetics: 1.07 (0.87, 1.32) vs insulin users: 0.78 (0.73, 0.83) vs SU users: 0.97 (0.87, 1.09) MI: 0.63 (0.28, 1.42) Stroke: 0.70 (0.53, 0.93)	Individuals taking metformin had a significantly lower rate of all-cause mortality compared to non-diabetic general population, non-metformin diabetic controls, insulin users and SU users. The sole exception was individuals whose diabetes was controlled with diet only. CVD incidence was also reduced in metformin users compared to non-metformin controls and insulin users but not compared to diet-controlled diabetics and SU users.
Selvin et al./*Arch Intern Med*./2008 [50]	40 (RCTs only)	Cardiovascular outcomes of oral GLTs	RCTs of oral GLTs (We report metformin vs any comparator here)	All-cause mortality: 0.81 (0.60, 1.08) Cardiovascular mortality: 0.74 (0.62, 0.89) Cardiovascular morbidity: 0.85 (0.69, 1.05)	Compared to other active comparators, metformin reduced cardiovascular death; other results not significant.
Crowley et al./*Ann Intern Med.*/2017 [51]	17 (observational studies)	Outcomes of metformin in populations with CHF, CKD, CLD	Non-metformin therapies	All-cause mortality CHF individuals: 0.78 (0.71, 0.87) CKD individuals: 0.77 (0.61, 0.97) CLD individuals (low risk-of-bias study): 0.43 (0.24, 0.78) Cardiovascular mortality in CHF individuals: 0.77 (0.53, 1.12)	Metformin use in patients with moderate CKD, CHF, CLD was associated with improvements in all-cause mortality.

Abbreviations: []—Number within brackets denotes reference within the main text, 95% CI—95% Confidence Interval, CAD—Coronary Artery Disease, CHF—Congestive Heart Failure, CKD—Chronic Kidney Disease, CLD—Chronic Liver Disease, CVD—Cardiovascular Disease, GLT—Glucose-Lowering Therapy, HF—Heart Failure, HR—Hazard Ratio, MI—Myocardial Infarction, OR—Odds Ratio, RCTs—Randomised Controlled Trials, SU—Sulfonylurea.

**Table 2:** Average 1-month cost of commonly used GLTs assuming frequently/maximum prescribed dosages.

GLT	Dose	Cost per Month
Metformin (standard formulation)	1 g twice a day	£7.08
Metformin slow release formulation	1 g twice a day	£6.40
Pioglitazone	45 mg once a day	£2.35
Gliclazide (standard formulation)	160 mg twice a day	£4.88
Sitagliptin (Januvia^®^)	100 mg once a day	£33.26
Linagliptin (Trajenta^®^)	5 mg once a day	£33.26
Canagliflozin (Invokana^®^)	300 mg once a day	£39.20
Empagliflozin (Jardiance^®^)	25 mg once a day	£36.59
Dapagliflozin (Forxiga^®^)	10 mg once a day	£36.59
Lirglutide (Victoza^®^)	1.2 mg once a day	£78.48
Semaglutide (Ozempic^®^)	1.0 mg once a week	£73.25
Dulaglutide (Trulicity^®^)	1.5 mg once a week	£73.25

Source: https://bnf.nice.org.uk/ (accessed on 22 October 2020).
